# Exploration of Reproductive Health Apps’ Data Privacy Policies and the Risks Posed to Users: Qualitative Content Analysis

**DOI:** 10.2196/51517

**Published:** 2025-03-05

**Authors:** Nina Zadushlivy, Rizwana Biviji, Karmen S Williams

**Affiliations:** 1 Department of Epidemiology and Biostatistics Graduate School of Public Health and Health Policy City University of New York New York, NY United States; 2 College of Health Solutions Arizona State University Phoenix, AZ United States; 3 Department of Health Policy and Management Graduate School of Public Health and Health Policy City University of New York New York, NY United States

**Keywords:** data privacy policy, reproductive health apps, Transparency, Health Content, Excellent Technical Content, Security/Privacy, Usability, Subjective, THESIS, THESIS evaluation, women’s health, menstrual health, mobile health, mHealth, menstruating persons’ health, mobile phone

## Abstract

**Background:**

Mobile health apps often require the collection of identifiable information. Subsequently, this places users at significant risk of privacy breaches when the data are misused or not adequately stored and secured. These issues are especially concerning for users of reproductive health apps in the United States as protection of sensitive user information is affected by shifting governmental regulations such as the overruling of *Roe v Wade* and varying state-level abortion laws. Limited studies have analyzed the data privacy policies of these apps and considered the safety issues associated with a lack of user transparency and protection.

**Objective:**

This study aimed to evaluate popular reproductive health apps, assess their individual privacy policies, analyze federal and state data privacy laws governing these apps in the United States and the European Union (EU), and recommend best practices for users and app developers to ensure user data safety.

**Methods:**

In total, 4 popular reproductive health apps—Clue, Flo, Period Tracker by GP Apps, and Stardust—as identified from multiple web sources were selected through convenience sampling. This selection ensured equal representation of apps based in the United States and the EU, facilitating a comparative analysis of data safety practices under differing privacy laws. A qualitative content analysis of the apps and a review of the literature on data use policies, governmental data privacy regulations, and best practices for mobile app data privacy were conducted between January 2023 and July 2023. The apps were downloaded and systematically evaluated using the Transparency, Health Content, Excellent Technical Content, Security/Privacy, Usability, Subjective (THESIS) evaluation tool to assess their privacy and security practices.

**Results:**

The overall privacy and security scores for the EU-based apps, Clue and Flo, were both 3.5 of 5. In contrast, the US-based apps, Period Tracker by GP Apps and Stardust, received scores of 2 and 4.5, respectively. Major concerns regarding privacy and data security primarily involved the apps’ use of IP address tracking and the involvement of third parties for advertising and marketing purposes, as well as the potential misuse of data.

**Conclusions:**

Currently, user expectations for data privacy in reproductive health apps are not being met. Despite stricter privacy policies, particularly with state-specific adaptations, apps must be transparent about data storage and third-party sharing even if just for marketing or analytical purposes. Given the sensitivity of reproductive health data and recent state restrictions on abortion, apps should minimize data collection, exceed encryption and anonymization standards, and reduce IP address tracking to better protect users.

## Introduction

### Background

The rights of menstruating persons in the United States have been at the center of major midcentury movements throughout history, whether that has been equal voting rights, safe working conditions ignited by the events of the Triangle Shirtwaist Factory fire, and freedom over their body autonomy. However, these rights and freedoms were further compromised in the 2022 *Dobbs v Jackson Women’s Health Organization* case, where the US Supreme Court overturned the 1973 *Roe v Wade* ruling, which guaranteed a constitutional right to abortion [1-3]. One year after the overruling, the nation’s perspective on reproductive health and the practice of collecting data through mobile apps designed to manage reproductive health have both deteriorated. Not only has the overruling threatened menstruating persons’ rights over their own bodies in terms of pregnancy, but it has also posed a risk to the safety of the information that menstruating persons share on such digital platforms.

The emergence of mobile health (mHealth) apps, coined as such by Robert Istepanian in 2008, has drastically shifted the way in which society approaches disease diagnosing, maintenance, monitoring, and management [4]. Such a shift has eased travel times and made selection of health care providers easier. While it was previously necessary to schedule physicians’ appointments to measure the health of an athlete or a patient with prediabetes, that is all simply done now through wearable devices such as Fitbit and Apple Watch or at-home glucose monitors that automatically update and transfer data from the user to the health care provider and alert users of irregularities detected, such as lowered oxygen levels and heightened heart rates. Due to the ease of use, users are increasingly turning to digital technologies such as mHealth apps for their health information and monitoring needs. However, smartphone technology use has been found to vary between nonmenstruating and menstruating persons, with 51% of nonmenstruating and 56% of menstruating persons using smartphones [5]. This trend is also reflected in health app use, with menstruating persons being more than twice as likely as nonmenstruating persons to turn to these apps for health information [5]. Over the past 6 years, the number of downloads for health and fitness apps alone has increased from over 1 billion in 2017 to 4.2 billion in 2023 [6].

This steady increase has also been observed with period trackers and reproductive health apps in particular, with a 2019 Kaiser Family Foundation study counting a third of American menstruating persons as users [7]. Additionally, in a study conducted among 360 health care providers, approximately 33% referred or provided information to their patients about sexual or reproductive health apps, and approximately 41% of those health care providers were confident and informed about sexual health apps [8]. Although there is continual discussion regarding security and confidentiality, the health care professionals indicated that there are potential benefits of sexual and reproductive health apps, such as being available to inform and assist underserved populations, engagement with and building of communities, training and education, and other health benefits. Reasons for use of sexual and reproductive health apps vary from menstruation monitoring, such as symptoms, changes, possible concerns, and cycle predictability, to fertility and conception tracking (ovulation) and organization of reproductive health information to pass on to health professionals during annual visits [9]. Such use has also allowed most menstruating persons to feel prepared, informed, and in control of their menstruation cycle in comparison to antiquated ways of tracking such as calendar markings [10]. Despite their ease of use and associated benefits, many apps rely on common yet sometimes inefficient methods to ensure that users read the terms and conditions and privacy policies. For example, the clickwrap (click-accept) approach, as observed in many apps, does little to ensure that users are actively reading the terms of use of each app. While there is no guarantee that users will read these terms, other approaches such as the scrollwrap (scrolling to the bottom to accept) approach may be better suited for mobile apps that deal with more sensitive data so that users are more inclined to read through highlighted areas and make educated decisions.

The convenience of reproductive health apps has posed challenges to users. Following the leak of the Supreme Court overruling draft in May 2022, many menstruating persons were left questioning what apps were safe to use. This led to an influx of users switching to reproductive health apps with more promising data privacy policies and practices, such as Clue and Stardust [11-13]. Given the sensitive information that these apps collect and store, in addition to recent shifts in the Supreme Court’s response to reproductive rights, the safety, confidentiality, and privacy of menstruating persons have taken center stage as potential risks associated with data misuse have become a growing concern [14]. These potential risks have become even more prominent in light of lapses in user policies designed to protect consumers and manage the flow of their data (data collection, data sharing, and data monitoring). Those in historically inequitable health situations, such as low-income, rural, and Black and other minoritized communities, have an increased risk of both using health apps as supplements for access to quality health care and being targeted and criminalized for seeking certain types of reproductive health care [15,16]. Professionals in the field, including legal scholars from the American Bar Association, have also expressed their concern about the potential misuse of advertising data [17,18]. In addition, while there has been misinformation regarding the misuse of private reproductive health data, it is vital for users of these apps to know and understand what data are collected, how they may be used, which companies may not meet their expectations and promises, and how they can protect their sensitive reproductive data by choosing apps that prioritize consumer safety [18].

### Policy Evaluations and Legal Framework

Although there are currently no comprehensive federal laws governing data privacy in the United States, certain case proceedings, agencies, and constitutional protections—such as the Health Insurance Portability and Accountability Act (HIPAA) Privacy Rule [19], the Fourth Amendment [20], the Food and Drug Administration [21] monitoring of medical devices, and the Federal Trade Commission (FTC) [22]—aim to protect individual privacy and medical records associated with identifiable health information. However, there is a lack of clarity regarding how these guidelines extend to mHealth apps and internet resources; for example, HIPAA does not restrict how apps may use health information that has been disclosed pursuant to the user’s right of access and how certain court rulings apply to and ensure privacy in personal, home, or public settings. In addition, privacy policies and data protections vary depending on an app’s country of origin. For instance, the European Union (EU) enforces comprehensive data privacy laws, providing a robust framework for identifying gaps in existing user data protection and highlighting areas for potential improvements. A comparative view of US and EU data privacy laws is presented in Multimedia Appendix 1 [19-37].

Although many apps require users to input identifiable information, certain users are at risk when such information is misused or not properly stored, secured, or shared. With reproductive health apps in the United States, the sensitive information required from users, coupled with recent legislative changes such as the overruling of *Roe v Wade*, underscore the importance of assessing the privacy policies and practices of sexual and reproductive health apps. So far, limited studies have analyzed the data privacy policies of these apps and addressed the safety issues associated with insufficient user protection. Therefore, this study aimed to (1) evaluate popular reproductive health apps and their respective data privacy policies, (2) compare data privacy laws governing these apps in the United States and the EU, and (3) recommend best practices for users and app developers to ensure user data safety.

## Methods

### Study Design

We conducted a qualitative content analysis between January 2023 and July 2023 on reproductive health apps available on the Apple App Store and Google Play Store and their publicly available privacy policies. The Transparency, Health Content, Excellent Technical Content, Security/Privacy, Usability, Subjective (THESIS) evaluation tool was used to assess the privacy policies and user agreements of the apps and compare the data privacy laws governing these apps in the United States and the EU [38].

### Ethical Considerations

This project did not involve human participants, and the City University of New York Graduate School of Public Health and Health Policy determined that it is not considered human participant research and, therefore, an institutional review board review was not required.

### App Selection

To identify apps that would offer the most comprehensive insights into data protection, we conducted a Google search for the leading apps available on both the Apple App Store and Google Play Store. The most commonly mentioned and popular reproductive health apps included Clue [39], Flo [40], and Period Tracker by GP Apps [41]. These selections were informed by recommendations from the *Women’s Health* magazine, which based its choices on obstetrician and gynecologist recommendations, and Healthline, which considered customer ratings and reviews, price, availability and accessibility, tools, and features [42,43]. Furthermore, we included a convenience selection with Stardust, which experienced a surge in users and became one of the most downloaded reproductive health apps following the *Roe v Wade* overruling due to its promise of data security using end-to-end encryption [44]. These 4 apps also appeared in the top 15 search results in both app stores based on their consumer ratings, reviews, and download numbers. Consumer ratings, reviews, and app downloads determine the popularity and acceptability of and satisfaction with the apps [45]. Furthermore, these 4 apps were specifically chosen because they provide a good understanding of how policies differ between the United States and the EU and, subsequently, how that affects app data use practices in the 2 regions.

The term *reproductive health app* is used consistently throughout this paper to refer to this category of mobile apps. The selected apps, along with others cited in previous sources, allow users to not only track menstruation cycles but also monitor pregnancy and sexual activity. The data stored on these apps are more sensitive than those stored on other mHealth apps as they often include email addresses, age, name, and date of birth. This sensitivity increases the risk to users if the data are shared or improperly stored.

Data were gathered from the Google Play Store and Apple App Store platforms for each app. App characteristics, including reviews, content rating, and update frequency, were evaluated on both iOS (Apple) and Android systems to identify any differences (Table 1). Notably, certain information is platform specific; for instance, the number of downloads is available only for Android users, whereas app rankings are accessible only to iOS users. This assessment aimed to determine whether apps regularly update their practices in response to the evolving landscape of privacy laws. App characteristics were initially evaluated in May 2023 and updated again in July 2023. It should be noted that these characteristics are subject to change.

**Table 1:** Characteristics of the Clue, Flo, Period Tracker by GP Apps, and Stardust [46] reproductive health apps evaluated on the Google Play Store and Apple App Store in July 2023.

			Clue		Flo		Period Tracker by GP Apps		Stardust
App developer			BioWink GmbH		Flo Health UK Limited		GP Apps		Stardust App LLC
Country of origin			EU^a^ (DE^b^)		EU (United Kingdom)		United States		United States
**Apple App Store**									
	Price	Free (additional pricing for in-app purchases)		Free (additional pricing for in-app purchases)		Free (additional pricing for in-app purchases)		Free (additional pricing for in-app purchases)	
	Release date	—^c^		—		—		—	
	Number of downloads as of July 2023	—		—		—		—	
	User ratings as of July 2023	4.8 out of 5 (343,848 reviews)		4.8 out of 5 (1,114,759 reviews)		4.8 out of 5 (61,896 reviews)		4.4 out of 5 (17,639 reviews)	
	Primary category or genre	Number 96 in “Health & Fitness” category		Number 7 in “Health & Fitness” category		Number 118 in “Health & Fitness” category		Number 195 in “Health & Fitness” category	
	Last updated as of July 2023	July 20, 2023		July 21, 2023		June 2022 (specific date unavailable)		July 19, 2023	
	Content (age) rating	12+		12+		12+		4+	
	In-app purchasing	Yes		Yes		Yes		Yes	
	In-app advertisements	Yes		Yes		Yes		Yes	
**Google Play Store**									
	Release date	October 9, 2014		April 12, 2016		October 7, 2011		June 27, 2022	
	Price	Free (additional pricing for in-app purchases)		Free (additional pricing for in-app purchases)		Free (additional pricing for in-app purchases)		Free (additional pricing for in-app purchases)	
	Number of downloads as of June 2023	≥10 million		≥100 million		≥10 million		≥50,000	
	User ratings as of July 2023	4.4 out of 5 (1.2 million reviews)		4.6 out of 5 (3.12 million reviews)		4.6 out of 5 (360,000 reviews)		2.9 out of 5 (26,400 reviews)	
	Primary category or genre	“Health & Fitness” category: specific ranking not available		10th highest-grossing app in “Health & Fitness” category		“Health & Fitness” category: specific ranking not available		“Health & Fitness” category: specific ranking not available	
	Last updated as of July 2023	July 24, 2023		July 26, 2023		September 19, 2022		July 20, 2023	
	Content (age) rating	Everyone		Everyone		Everyone		Everyone	
	In-app purchasing	Yes		Yes		Yes		Yes	
	In-app advertisements	Yes		Yes		Yes		Yes	

^a^EU: European Union.

^b^DE: Germany.

^c^Not available.

### Downloading, Registering, and Using

**Figure 1:**
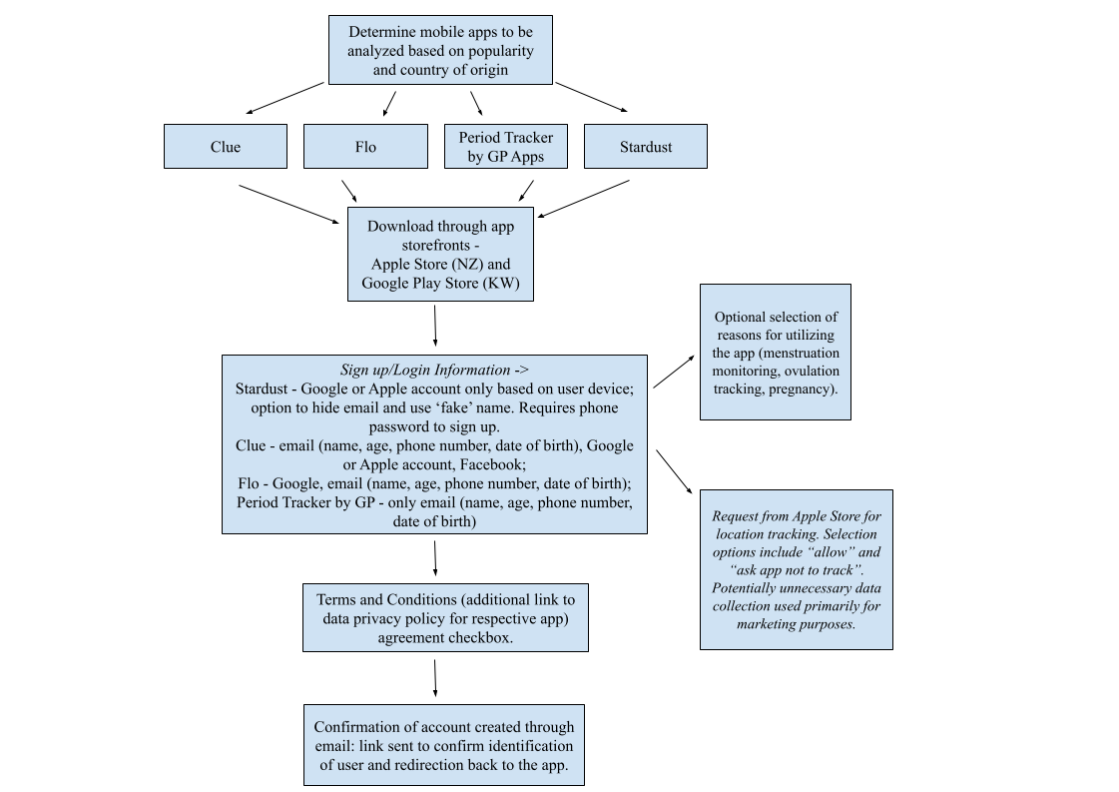
Visualization of the reproductive health app selection and the registration on each app between May 2023 and July 2023.

In total, 2 researchers (NZ and KSW) downloaded and registered on all 4 apps (Clue, Flo, Period Tracker by GP Apps, and Stardust) using both an iOS and Android system between May 2023 and July 2023. All apps had a “log in” and “create an account” option that used an email and password or an existing Google or Facebook account and a passive or explicit agreement with the privacy policy and terms of service. The passive agreements had language such as the following—“by creating an account you are agreeing...”—whereas explicit agreements required a button to select “agree” before continuing with the registration. These steps can be observed in Figure 1. Enrollment flow varied between apps, with common information required for signing up being email, age, date of birth, and phone number.

### Data Analysis: THESIS Evaluation Tool

To properly assess the reproductive health apps chosen, particularly regarding their privacy and security, it was important to find an evaluation tool that addressed these qualities. Unlike the commonly used Mobile App Rating Scale assessment tool that rates apps based on subscales, items, descriptors, and anchors, the THESIS evaluation tool dedicates one of its domains specifically to app privacy and security [47]. The THESIS evaluation tool consists of 6 domains on which an app is rated on a scale from 1, the lowest, to 5, the highest: transparency, health content, technical content, security and privacy, usability, and subjective rating. Due to the objective of this research being the security of user data, the security and privacy domain, consisting of 6 subcategories, was the part of the evaluation tool used. The subcategories and their rankings are shown in Textbox 1.

**Subcategories of the security and privacy domain of the transparency, health content, excellent technical content, security and privacy, usability, and subjective evaluation tool and their rankings.**

- Protection against theft and viruses: does the app follow best practices in security with optimal antivirus and safeguards against breaches? This is found in the privacy policy of an app.
- Authentication: is the authentication procedure optimal? User experience is based on receiving an email or SMS text message notifications for creating an account, in addition to password protection for accessing the user’s profile.
- Data sharing: when sharing information, does the app use best practices? This is found in the privacy policy of an app.
- Maintenance: does the app have regular cycles to update and patch its security? This is found in the Apple App Store or Google Play Store under “version history” for each app—last update occurred during the month of rating or the month before (the update schedule is very consistent; rating of 5), last update occurred 2 months before the time of rating (the update schedule is generally consistent; rating of 4), last update occurred between 3 and 5 months before the time of rating (the update schedule is a little inconsistent; rating of 3), last update occurred between 6 months and a year before the time of rating (the update schedule is completely inconsistent; rating of 2), and last update occurred >1 year before the time of rating (rating of 1).
- Signaling of breaches: if a breach occurs, does the app have a method to notify its users? This is found in the privacy policy or the terms and conditions of an app.
- Anonymization: does the app appropriately anonymize individuals? This is found in the privacy policy or the terms and conditions of an app [38].
  To note, the Federal Trade Commission (FTC) has expressed concern regarding the legitimacy of data anonymization as promised by multiple apps, not just reproductive health apps, calling claims as potentially deceptive trade practice and violating the FTC Act when untrue. Significant research has shown that anonymized data can often be reidentified, especially in the context of location data. One set of researchers demonstrated that, in some instances, it was possible to uniquely identify 95% of a dataset of 1.5 million individuals using 4 location points with time stamps [48].

The evaluation tool includes 2 additional columns dedicated to rationalizing the pros and cons of specific ratings. The Why Not −1 column indicates why points were not deducted from the app’s score, whereas the Why Not +1 column indicates why points were deducted from the final score.

## Results

### Reproductive Health App Privacy Policy Summaries

With 1.8 million mobile apps available on the Apple App Store and 2 million available on the Google Play Store, both platforms have released their own guidelines for developers seeking to launch their apps [49,50]. The Apple App Review Guidelines have a specific section dedicated to privacy policy requirements, stating that all apps must provide their users with a privacy policy either within the app or as a link when developing for iOS. These privacy policies must “identify what data, if any, the app or service collects, how it collects and uses that data, confirm that any third party with whom an app shares user data (in compliance with these Guidelines)—such as analytics tools, advertising networks and third-party SDKs, as well as any parent, subsidiary or other related entities that will have access to user data—will provide the same or equal protection of user data as stated in the app’s privacy policy and required by these Guidelines, and explain its data retention/deletion policies and describe how a user can revoke consent and/or request deletion of the user’s data” [51]. These guidelines also include more information on secure user consent, data minimization, and data collection. The Google Play Store guidelines are similar, posted on the Google Play Console Help Center, and require developers to state how private user data are collected, protected, and handled. However, the expansion of the privacy policy requirement to all mobile apps released through the Google Play Store is recent, as of 2022 [52]. In addition to this, in July 2021, the Google Play Store began requiring apps to fill out a data safety form that needs to align with the apps’ actual data use. That being said, the only Google Play Store requirements are that information such as where the data are sold to and how they are shared be stated in the data safety form. On the basis of this information, Google does not clearly state its concern for the privacy of its users. The same pattern was observed on the Apple App Store, with the only difference being Apple’s location tracking option that pops up with every app downloaded. Because of these guidelines, the 4 reproductive health apps evaluated have their own data privacy policies separate from their terms and conditions that must be agreed to before use of the app.

Multimedia Appendix 2 [39-41,46] shows the analysis of each reproductive health app regarding its privacy policy, the type of data collected, how they are protected (varying types of encryption vs none at all to store sensitive data and protect them from being stolen, changed, or compromised), who they are shared with, what options are available for users seeking to delete or opt out of data sharing, and potential concerns that remain. In addition to this, a column was dedicated to assessing the accessibility of privacy policies to users, which often affects the probability of users reading through them rather than simply clicking on Agree to access the app functions, otherwise known as the clickwrap approach. Furthermore, both anonymization and end-to-end encryption are included in one section because they are used with differing data, such as anonymization for metadata such as IP addresses and end-to-end encryption for data input into the app by the user, and, thus, vary in data protection. Assessment of these apps was first conducted at the beginning of the research in November 2022 and then updated twice, once in May 2023 and again in July 2023 for the most up-to-date assessment. The final column of the table addresses concerns related to the methodology used by each app.

### THESIS Evaluation Tool

After analysis of individual app policies, the THESIS evaluation tool allowed for a thorough comparison of the security and privacy of the 4 mobile apps selected. The tool was first used to assess the EU apps, Clue (Table 2) and Flo (Table 3), with Clue being the first due to its earlier release date, followed by the assessment of the US app Period Tracker by GP Apps first due to its earlier release date and then of Stardust as that is the most recently released app evaluated. This chronological order allowed for a better understanding of the changes made in the app privacy policies with the changing landscape of reproductive health rights. Scoring was based on an initial analysis of the apps in May 2023 that was repeated in July 2023 to see whether any changes were made as more state privacy laws were introduced. A description is provided for every score to explain the reasoning behind each grade. The subcategories are those of the THESIS evaluation tool and its score guide.

**Table 2:** Transparency, health content, excellent technical content, security and privacy, usability, and subjective evaluation of Clue, a European Union (EU) app that falls under General Data Protection Regulation protections, based on an initial analysis of the app in May 2023 repeated in July 2023. Score: 3.5 for the security and privacy domain.

Subcategory	Score	Why not –1	Why not +1
Protection against theft and viruses	1	—^a^	Not enough information available anywhere in policies or documents
Authentication	4	For verification, the app sends an email to the address used for registration, and the user is unable to input information into the app calendar without this verification; PIN^b^, Touch ID (iPhone 5S-8), or Face ID (iPhone X-13) authentication is available and is intended to automatically encrypt Clue data and prevent someone else from accessing users’ information through a user’s device	Information on this feature is not explicitly stated; requires a look through the privacy policy on the app’s website.
Data sharing	3	Data-sharing practices seem to be more transparent than those observed on other apps. The creators make it a point to state that privacy is one of their top priorities. It also follows guidelines of EU data privacy laws, which are said to be the strictest in the world.	However, Apple has its own ranking of data based on the analytics the app creators provide them with. This portion is contradictory as it states the following: “Data linked to you may be collected and linked to your identity: contact info, health and fitness, location, etc” [53]. Data are also stored, which poses threats if subpoenaed.
Maintenance	5	The app is updated almost every week. The last update occurred 5 days before rating.	—
Signaling of breaches	1	—	Not enough information available anywhere in policies or documents
Anonymization	3	The app states not to use any identifiable information of the users for any purposes, including data analyses. Encryption is used.	IP address collection poses a threat to user safety. HTTP is not the safest encryption method and “the data that is transferred over an HTTP connection is not encrypted, so you run the risk of third-party attackers stealing the information” [54].

^a^None.

^b^PIN: personal identification number.

**Table 3:** Transparency, health content, excellent technical content, security and privacy, usability, and subjective evaluation of the Flo app data use practices in July 2023. Score: 3.5 for the security and privacy domain.

Subcategory	Score	Why not –1	Why not +1
Protection against theft and viruses	4	General information provided, such as an email for the data protection officer. The website mentions vulnerability testing and system monitoring or alerting.	Difficult to test accuracy in real-life settings unless issues arise.
Authentication	4	The app sends an email to the address used for signing up for verification, and the user is unable to input information into the app calendar without this verification. There is an option to password protect the app to enable secure access.	Secure access is not a pre–set up option; users have to know how to access it and turn it on. This is only mentioned in the app’s web page rather than being originally included in the app.
Data sharing	2	At first glance, it looks good, and data are not said to be sold to other companies. A user’s cycle can be linked to other apps. There is an emphasis on sharing rather than selling. The data stored are also encrypted.	It is said that personal data may be processed in relation to the company’s interests in providing services, commercial interests, and “wider societal benefits.” The app connects with AppsFlyer for marketing and promotions and requires consent, but consent is required to make an account regardless. This involves a lot of technical identifiers. The “security of personal data” section is contradictory as it says that the data are encrypted [40]. It is important to note that encryption does not mean that data are not accessible by the company doing the encryption. It just means that the data do not reside on their servers in plain-text format. Use of the term “encrypted” gives the impression that the company cannot see the data, whereas in reality, it just means that the data are not stored in a plain-text format.
Maintenance	5	The app is updated almost every week. The last update occurred 5 days before rating.	—^a^
Signaling of breaches	4	There is a separate section in the policy for security breaches and what steps the app will take if that occurs. A notification is said to be sent out, and the app will take steps such as deleting user information, resetting passwords, and logging out of user devices if such a breach occurs.	Does not seem to be responsible for third-party data breaches (although it states otherwise) given a previous data breach with third-party Facebook Analytics tool despite existing safeguards.
Anonymization	3	The app states not to sell any identifiable information. The app also gives the option to be used in anonymous mode. There is an option to not allow the app to track Apple Health or Fitbit statistics. All data are encrypted using KMS^b^ [55].	IP address and unique device identifiers (IDFA^c^) are collected. The Apple App Store also has contradictory information saying that data are linked to a user, such as location, contact information, identifiers, user content, and sensitive information.

^a^None.

^b^KMS: Key Management Service.

^c^IDFA: identifier for advertisers.

Flo users in the United States are subject to US laws, but the company must also comply with EU laws for EU-based users. In Table 3, scoring was based on an initial analysis of the app that was repeated 2 months later to see whether any changes were made as more state privacy laws were introduced. A description is provided for every score to explain the reasoning behind each grade. The subcategories are those of the THESIS evaluation tool and its score guide. A major concern is the app’s use of IP address tracking as well as the use of data by third parties for advertising and marketing.

Period Tracker by GP Apps (Table 4) is a US-based app potentially required to adhere to the General Data Protection Regulation because of its European users. Scoring was based on an initial analysis of the app in May 2023 that was repeated in July 2023 to assess changes as more state privacy laws were introduced. A description is provided for every score to explain the reasoning behind each grade. The subcategories are those of the THESIS evaluation tool and its score guide, as seen in the previous 2 evaluations.

**Table 4:** Transparency, health content, excellent technical content, security and privacy, usability, and subjective evaluation of Period Tracker by GP Apps’ data use practices in July 2023. Score: 2 for the security and privacy domain.

Subcategory	Score	Why not –1	Why not +1
Protection against theft and viruses	2	Some security is listed on the app’s website, such as limiting access to information to authorized employees and contractors.	Does not seem to be held accountable for any breaches or collection of data that may occur.
Authentication	3	Option to turn on finger ID or passcode is available.	It is not clearly stated where this option is and how to actually use it.
Data sharing	2	—^a^	Limited information available, which makes it seem even more as though the app does not want to disclose its privacy and data sharing information. Nothing about unidentifiable information or how sharing occurs is noted.
Maintenance	2	—	Last update and bug fix was 6 months before rating. Seems to be an update pattern based on the version history available on the app store.
Signaling of breaches	2	It does say that users will be informed of any changes occurring within the system; this may mean breaches as well.	No information on breaches is available other than the app stating that they are possible and they are not responsible.
Anonymization	2	Option for using without an account is possible but extremely limited.	Nothing else is noted about anonymization from the app itself. However, the app store does say that one can disable their information being used by third parties, but this is written in tiny letters on the website.

^a^None.

The final app evaluated was Stardust (Table 5), an app that underwent internal data storage and use and account creation modifications as a response to *Dobbs v Jackson* and the wave of worry among menstruating persons using reproductive health apps [56]. Stardust was purposely chosen because of its recent emergence and emphasis on user data protection and different account creation practices. Stardust emphasizes end-to-end encryption and minimal data collection. Table 5 scoring was based on an initial analysis of the app in May 2023 that was repeated in July 2023.

**Table 5:** Transparency, health content, excellent technical content, security and privacy, usability, and subjective evaluation of the Stardust app’s data use practices in July 2023. Score: 4.5 for the security and privacy domain.

Subcategory	Score	Why not –1	Why not +1
Protection against theft and viruses	3	Some information regarding theft was provided.	General statement: “We take all reasonable and appropriate measures (including encryptions) to protect all collected Personal Data from loss, theft, misuse, and unauthorized access” [46].
Authentication	5	The app sends an email to the address used for signing up for verification, and the user is unable to input information into the app calendar without this verification. Uses Rownd, a privacy-first authentication platform.	—^a^
Data sharing	4	Data-sharing practices seem to be more transparent than those observed on other apps, closer to what is observed in the EU^b^. Data are said to be encrypted.	However, data are still said to be potentially provided if required by government agencies. It is not clear what that includes.
Maintenance	5	The app is updated almost every week. The last update was 1 day before rating.	—
Signaling of breaches	1	—	No information available anywhere in policies or documents
Anonymization	5	End-to-end encryption that is only accessible to the user. The policy states to also not track or use IP addresses. Even if the government asks for the information, it should not be linked to a specific user. Users are also able to come up with their own username and are not required to use their legal name.	—

^a^None.

^b^EU: European Union.

### App Store Information Discrepancies

**Figure 2:**
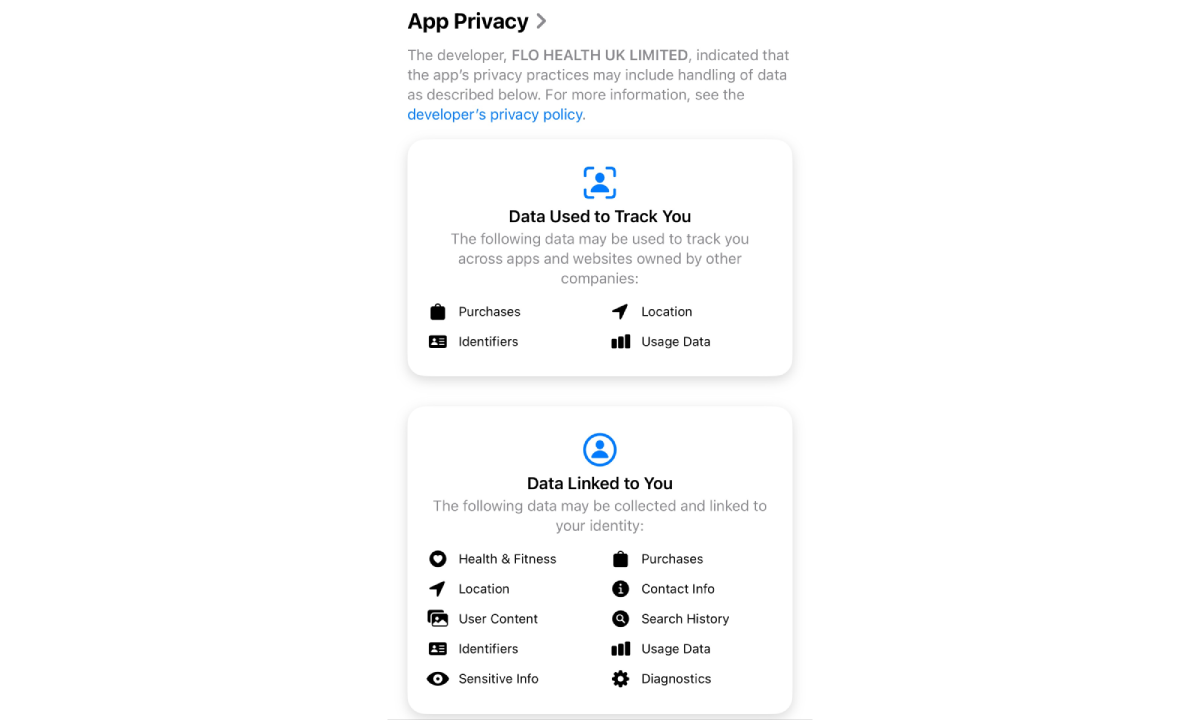
Apple App Store description of Flo data collection and sharing.

Another important aspect to consider are the discrepancies observed between data collection information on the Apple App Store and on the apps themselves. Despite app promises of data privacy, there are notable differences between their respective privacy policies and what is stated in the Apple App Store. As shown in Figure 2 [57], the Apple App Store included its own description of potential data collection based on each app. While Flo has a history of violating the privacy policy promised to its users, the data that may be collected as listed by the Apple App Store are different from those listed in the app’s privacy policy.

This is in comparison to the app privacy summary of Stardust, which more closely aligns with the information provided in the app’s own privacy policy.

**Figure 3:**
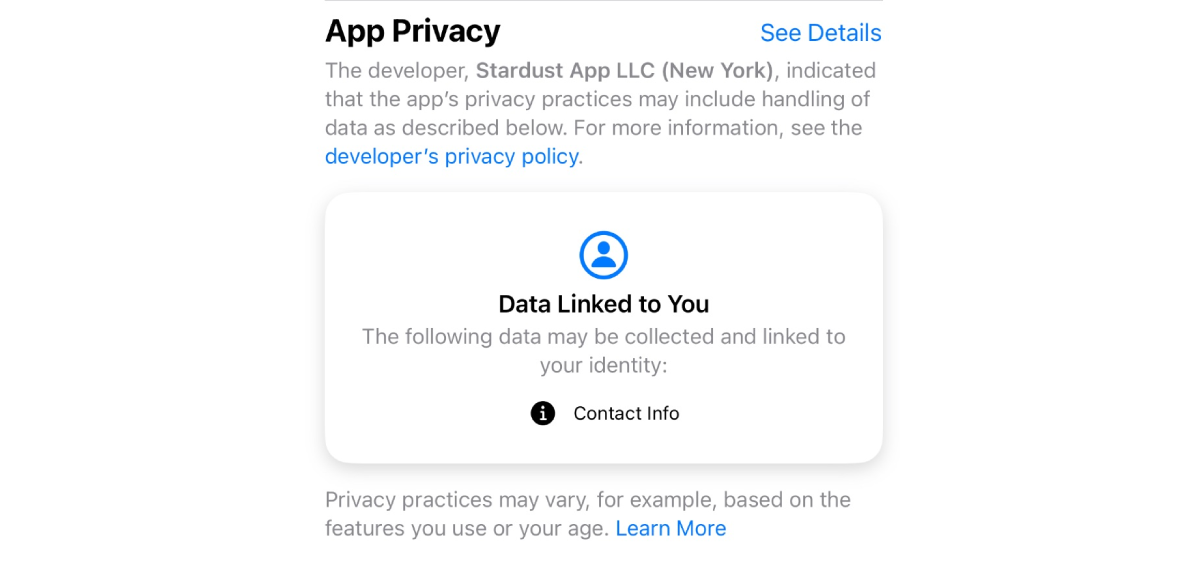
Apple App Store description of Stardust data collection and sharing for comparison with that of Flo [39].

Such discrepancies make it difficult for users to determine what data are being collected and shared and, thus, cause confusion when deciding what reproductive health app should be used. These discrepancies are also reflected in Figures 2 and 3 analyzing the apps, where contradictory information alters app ratings.

In addition, another notable discrepancy is the content rating for the apps as observed on the Apple App Store and Google Play Store. While 75% (3/4) of the apps have ratings of 12+ on the Apple App Store, all 4 apps are rated E for Everyone on the Google Play Store. Given the content of the apps and the sensitivity of the data provided, the Apple App Store rating should be considered the more suitable one. It is interesting to note that a statement on the Google Play Store mentions that “content ratings on Google Play are provided by the International Age Rating Coalition (IARC) and are designed to help developers communicate locally relevant content ratings to users” and, thus, makes it difficult to understand what causes the discrepancy in content rating on both platforms [59].

## Discussion

### Principal Findings

This study evaluated popular reproductive health apps and their respective data privacy policies, compared data privacy laws governing these apps in the United States and the EU, and recommended best practices for users and app developers to ensure user data safety. The characteristics of reproductive health app data privacy, safety, and use were indicated. Using the THESIS evaluation tool, the privacy and security features of 4 reproductive health apps were identified, and the determined discrepancies in these features were described in the previous sections. Although there are successes and good practices in app user data management, potential concerns remain. Adopting best practices from multiple sources, such as end-to-end encryption, can help enhance the privacy and security of user data on reproductive health apps.

### Potential Concerns

A careful evaluation of the 4 selected reproductive health apps identified Stardust as the most privacy-centered app at the time of this qualitative content analysis. It should be noted that policies and data use practices are continually evolving as apps adapt to new requirements and guidelines. Although Stardust has lower ratings on both the Google Play Store and Apple App Store, which is attributed to its recent release and interface issues, it provides the most comprehensive data protection to its users. This is due to its limited data collection, such as excluding IP tracking; transparent privacy policy; specified encryption methods; options for deleting previously shared data; and the use of third-party platforms that assess deidentified data in a secure manner. While Clue, Flo, and Period Tracker by GP Apps collect IP address data, Stardust focuses on location data, such as country and time zone data, which minimizes the likelihood of connecting a user to a specific location and any reproductive health information. Furthermore, the app is transparent about the third parties it engages with and how user data are used. Although Flo and Clue use encryption to protect the confidentiality of the digital data stored across various platforms, the combination of IP address collection and the extensive use of third-party data brokers for analytics may pose unique risks to users, especially when linked with sensitive information such as missed periods and unique device identifiers [18].

When comparing the privacy and security of the data collected on all 4 apps—Clue, Flo, Period Tracker by GP Apps, and Stardust—determining what factors make one reproductive health app during this time a better and safer choice for users than others was critical. Although existing privacy policies attempt to combat the easy accessibility of user data and ensure stricter regulation on data sharing and breaches, the ultimate responsibility to ensure the safety of reproductive health information and app users falls on the app developers themselves. In this assessment, these factors included how accessible the privacy policy of each app is; what data are collected and how they are made secure; which, if applicable, third parties data are shared with and for what purposes; and the ease with which users may have their data deleted and opt out of future data collection.

In addition to this, a common trend among all apps was the questionable access to privacy policies and user agreements that does little to assure and encourage users to read through these agreements rather than selecting "Accept" just to gain access to the apps’ functions.

Despite identified potential concerns, for users in regions without strict abortion regulations, Clue and Flo may be safe choices considering that IP address tracking alone, without any other identifiable data-point tracking as the apps promise, should not pose a significant threat. For full protection, users should create anonymous accounts, as facilitated in Flo and anticipated in Stardust, and enable password protection where available. Most importantly, users are advised to avoid reproductive health apps such as Period Tracker by GP Apps due to its infrequent system updates, lack of accountability, inadequate encryption in data collection and sharing, and insufficient transparency in their privacy policies. Using an app that does little to protect individual users may make it prone to data breaches and risks data use for things that the user is not aware of, such as during subpoenas or other investigations.

### Comparison With Other Work

The findings of this study have also been supported by previous data privacy research, systematic reviews, and scoping reviews conducted in relation to numerous mHealth apps, including mental health, diabetes management, and physical activity tracking. Although they used different analysis methods from the THESIS evaluation tool, Benjumea et al [60] also concluded that the re-evaluation of the data collected on these apps, stated as “minimization” by the General Data Protection Regulation, is necessary to determine whether the scope of the personal information collected on these apps is beneficial to the users or may be limited to protect user data. In addition, the research conducted by Alfawzan et al [61] involving the 23 most popular mHealth apps for menstruating persons emphasized the threats associated with third-party data sharing, determining that 87% of the apps assessed were found to partake in such sharing and stating that “user consent, especially about sharing data in general or with a third party, is a concern for women’s privacy...research indicates that end users do not have full awareness of what their consent entails.” Furthermore, key findings of the analysis of reproductive health apps, including Flo, in the article “In Post Roe v Wade Era, Mozilla Labels 18 of 25 Popular Period and Pregnancy Tracking Tech With *Privacy Not Included Warning” by the Mozilla Foundation concluded that there is no clear stand on data sharing with law enforcement; password requirements are especially low; and there is an absurd amount of personal information being collected ranging from device IDs and IP addresses to pregnancy symptoms and physicians’ appointments [62].

Other articles, such as “Mobile Health and Fitness Apps: What Are the Privacy Risks” by Privacy Rights Clearinghouse, outline discussed concerns about data collection for advertising purposes, stating that “mobile applications, especially apps that you download for free, depend on advertising to make money...they may share personally identifiable information with advertisers, or allow ad networks to track you...if an application collects your universal device ID (UDID) or embeds a unique ID in the application you download, analytics data can be traced back to you personally” [63]. This concept closely relates to the accusations previously made against Flo regarding their user data sharing between 2016 and 2019.

### Importance of Encryption and Real-Life Application

Following the overruling of *Roe v Wade*, major reproductive health apps such as those assessed in this study have taken it upon themselves to work toward a safer way for menstruating persons to track, monitor, and maintain their reproductive health. With the introduction of end-to-end encryption in the late 90s, these apps have adopted a way to provide users with data privacy while also collecting enough user analytics to better their own apps [64]. Such encryption methods, as observed in Stardust, are meant to ensure that sensitive data such as personal health information are protected from unauthorized disclosure. “In [end-to-end encryption], the data is encrypted on the sender’s system or device, and only the intended recipient can decrypt it. As it travels to its destination, the message cannot be read or tampered with by an internet service provider (ISP), application service provider, hacker or any other entity or service” [65].

One of the issues that only Stardust addressed is the collection of IP addresses and location tracking. Despite end-to-end encryption dispersing and disconnecting user data from user profiles, IP location tracking and mobile ID tracking leads to the risk of connecting deidentified user information with specific locations and phone use. For example, if a user is located in a US state that has banned abortions, such as Alabama, Idaho, Tennessee, and Texas, there is a risk that state officials could obtain personal data that reveal pregnancy or potential termination of pregnancy based on period or symptom tracking [66-68]. In addition to this, the location data tracked through these apps could also reveal a user’s visits to abortion clinics via IP addresses. If state agencies receive a subpoena, they may obtain this information regarding users and the medical professionals providing banned procedures. In states where abortions are illegal, lack of proper data protection raises implications that could endanger users after the overruling of *Roe v Wade*, especially among those with historically inequitable health situations [69]. Increased risk of government officials legally obtaining personal information, even IP addresses that may directly link data points to an individual user, due to these laws has led to the assessment of the data safety provided by individual apps and what steps may be taken from the users’ and the developers’ side to ensure future security. In addition to this, some companies such as Flo and Clue have developed the option for users to create passwords to protect their app accounts through encryption methods that ensure access to the app only through the user’s phone. Analyzing the proper steps that Flo, Clue, and Stardust have taken to heighten their users’ safety allowed for the assessment of data privacy lapses in Period Tracker by GP Apps.

### Prioritizing Marketing Over User Protection

Evaluation of privacy policies also shed light on what role data collection plays apart from direct user experience improvement. For all the apps except Stardust, it is explicitly stated that some data are shared with third-party apps for marketing purposes, such as reaching potential users based on current user preferences. The issues associated with this are similar to what happened with Flo sharing its user information with Facebook Analytics services that resulted in a data breach. Flo’s statement on this data breach allows for insights on what user data have been used for recently—insights from Facebook Analytics found “that it’s challenging for [Flo] users to log childbirth and enter postpartum mode...This finding was driven by the insights from analytical systems, and with its help [Flo] managed to improve user experience and stressless” [70]. However, many, including the FTC, have speculated “...that Flo’s data-sharing practices had allowed third-party companies to use said personal health information expansively, including for advertising...” [71]. Apart from Flo, apps such as Clue also state that they use data for promotional purposes. With the sensitivity of the data at hand on these apps, reproductive health app developers may consider limiting their marketing resources and turning to internal analytics without the use of third parties unless third parties have their own practical user privacy tools that may be trusted and proper business associate agreements are in place.

### Recommendations

With the thorough analysis of the 4 most popular reproductive health apps available on both the Apple App Store and Google Play Store, it is possible to make recommendations for app developers, major app platforms, and individuals looking to use reproductive health apps.

First, to ensure user data safety, developers should prioritize the access to and reading ease of privacy policies. While users are currently required to agree to the terms and conditions of reproductive health apps before the use of the apps’ features, there is no guarantee that these policies are actually being read and not just bypassed. Although court cases such as *Bassett v Electronic Arts Inc* currently require terms and conditions to be clearly visible to users (ie, not hidden by fonts or an unclear location of the link), all the apps evaluated used the clickwrap approach for user privacy policy consent [67,72]. To make it more probable that users are actively reading the conditions they are agreeing to, using a scrollwrap approach, one that requires users to scroll through the entire policy agreement before checking off an “I accept” button that becomes active only once the scrolling is completed, is preferable [73]. With this method, app privacy policy writers should opt to highlight the most important aspects of the agreement in layman’s terms to capture the user’s attention. While this does not guarantee that users are actively reading what they are agreeing to, if information is highlighted, it may catch users’ attention better. App developers may also consider shifting to preset anonymous modes of user profiles, as observed as an option in Flo, as well as requiring virtual private network use to access the app functions to limit the chances of data being specifically linked to individual users.

Second, the evaluation of previously enacted policies also requires adaptation to the ever-changing field of technology that poses new threats to mHealth app users.

Third, users should be encouraged to make educated decisions when selecting reproductive health apps for personal tracking, monitoring, and management. Users should be aware of what data are collected; where those data go; and what risks arise if their personal information is shared, handled, or stored differently than what is expected or promised. This could be made possible by major app platforms such as the Apple App Store and Google Play Store closely monitoring the collection of data on reproductive health apps and making sure that apps are constantly updating their users on changes to data privacy settings, as well as adhering to data-sharing restrictions stated in individual app policies. Platforms should also consider altering their rating criteria, making data privacy one of the most crucial qualities of a highly rated app and, thus, increasing user health literacy and guiding them to the secure apps.

Finally, end-to-end encryption and data anonymization methods should both be used to protect not only the reproductive health data that users have provided to the apps but also the data collected by the apps themselves, including device IDs and IP addresses. Such practices would further ensure that deidentified personal health information may not be linked to a specific user while also providing developers with enough user analytics to continue updating the app’s functionality. Furthermore, it is vital for apps to follow anonymization rules set out by the FTC and other regulatory systems rather than make false promises that do little to protect user data.

### Strengths and Weaknesses

While many other studies have assessed the data protection aspects of reproductive health apps, this is one of the first that takes a comprehensive approach to data privacy, from the analysis of existing data privacy policies to individual encryption methods. In addition to this, having more than one researcher assess each individual app allowed for limitation of bias and increase in reliability and validity. However, there are a few weaknesses concerning this study. These include the lack of generalizability as only 4 apps were assessed, whereas there are >25 reproductive health apps on both the Google Play Store and Apple App Store platforms; the constant updates of Clue, Flo, and Stardust that change the way in which each protect, collect, and share data; and the ever-changing field of data privacy, with company statements and policies changing since the beginning of this study in 2022. Furthermore, a limitation is the convenience sampling conducted through which the 4 apps were chosen as there was no systematic search of apps conducted apart from that of country of origin for the 2 EU apps listed. Finally, what was true last year regarding these apps may not be true today. With the changing landscape of data privacy policies and federal and local governments changing platform requirements, these apps may have taken steps to further protect user data. In addition, most apps are constantly updating their own policies and interfaces.

### Conclusions

As stated by Ritesh Kotak, a Toronto cybersecurity and technology analyst quoted in CBC Canada’s “Americans are being urged to delete period tracking apps. Should Canadians do the same?” article, “When you downloaded that app, how much did you pay for it? What’s your monthly subscription fee? If the answer is zero, if you’re not paying for the product, then you are the product” [68]. Moving forward in a technology-based society heightens the risk of information sharing and data security violations, which becomes of increased concern when combined with reproductive health changes within the Supreme Court and local government agencies. Because of this, it is crucial for the landscape of data privacy protection to reflect the changes being made and prioritize the safety and protection of users. More studies emphasizing the use of data collected by reproductive health apps and long-term threats caused by the sharing of such data, in addition to lapses in outdated policies in place, will further assist users in making smart app selection decisions and encourage app developers to assert user data protection as their top priority. Reproductive health apps are meant to function as resources available to the general population designed to make their day-to-day lives and activities simpler; they are not meant to be guidelines for maternal or menstruating health and, thus, should be treated as such. That is why limiting the data collected by these apps and taking precautions to ensure data privacy is in the best interest of users and app developers.

## Appendix

Multimedia Appendix 1 A breakdown of existing policies in the United States and the European Union’s General Data Protection Regulation, in addition to their respective issues regarding reproductive health apps, organized in chronological order before conducting the app analysis in May 2023 for better understanding of how policy making has changed and shifted as technology becomes more prevalent.

Multimedia Appendix 2 A description of the privacy and data use practices of reproductive health apps from their respective data privacy policies collected in July 2023.

## Abbreviations

EU: European Union
FTC: Federal Trade Commission
HIPAA: Health Insurance Portability and Accountability Act
mHealth: mobile health
THESIS: Transparency, Health Content, Excellent Technical Content, Security/Privacy, Usability, Subjective

## Data Availability

Data sharing is not applicable to this paper as no datasets were generated or analyzed during this study.
