# Supplementary material for: Exploration of Reproductive Health Apps’ Data Privacy Policies and the Risks Posed to Users: Qualitative Content Analysis
Source: J Med Internet Res. 2025 Mar 5;27:e51517. doi: 10.2196/51517 (PMC11923453; doi:10.2196/51517)
Supplement: Multimedia Appendix 1 [file jmir_v27i1e51517_app1.docx]

This Appendix represents a breakdown of existing policies in the US as well as EUs General Data Protection Regulation, in addition to their respective issues for reproductive health app application. It is organized in chronological order prior to conducting app analysis in May 2023, for better understanding of how policy making has changed and shifted as technology became more prevalent.

| Act | Effective Since | Brief Description | Existing Gaps |
| --- | --- | --- | --- |
| *In the United States* |  |  |  |
| The Fourth Amendment | 1791 | Protects people from unreasonable searches and seizures by the government [1]. | Vague definition of reasonable searches. Leaves wiggle room for government agencies to obtain personal data from other sources, such as third party data brokers used by reproductive health apps. Not updated to fit current changes in electronic data. |
| The Federal Trade Commission Act (FTC) | 1914 | Requires companies not covered by HIPAA to adhere to their privacy and security claims, and report any breaches to them and users through the Health Breach Notification Rule. Even if companies do not make any privacy claims, security must be maintained based on the sensitivity of user data [2]. | Currently the best at spotting breaches of contracts pertaining to personal health information and requiring companies to notify users of breaches. However, it is uncertain how often the commission investigates reproductive health policies unless there is a complaint from users and/or third parties. The cases must also be approved by federal courts which in the case of reproductive health apps may side with government agencies. |
| The Food and Drug Administration (FDA) Medical Device Subsect | 1984 | Subsect that regulates the sale of medical device products in the U.S. and monitors the safety of all regulated medical products [3]. | Only refers to medical devices. Reproductive health apps often fall under the “software functions that are intended for individuals to log, record, track, evaluate, or make decisions or behavioral suggestions related to developing or maintaining general fitness, health or wellness” category. Reproductive health apps falling outside of this scope, such as those that can provide birth control, undergo very minimal clearing [4]. |
| The 1986 Stored Communications Act (SCA) | 1986 | Governs access to stored wire and electronic communications such as emails and other online messages held by service providers, including third parties with which user data is shared [5]. | Present day digital platforms and devices have “outpaced the law” outlined by the SCA, despite occasional additions and amendments to the law. The government holds control of the data meaning that if state agencies request private data of reproductive health apps, there is a chance that the SCA will support the state rather than the user [6]. |
| Children's Online Privacy Protection Rule  (COPPA)  ** Policy added due to differing app age requirements on the Apple App Store and Google Play Store* | 2000 | “Imposes certain requirements on operators of websites or online services directed to children under 13 years of age, and on operators of other websites or online services that have actual knowledge that they are collecting personal information online from a child under 13 years of age” [7]. | Often labeled as outdated because it was created before the introduction of smartphones and social media, and unconstitutional by some because it prevents users of that age from exercising their freedom of speech. Some platforms have independently raised their age restriction to 16 years old based on the data/information shared or collected [8]. |
| Health Insurance Portability and Accountability Act (HIPAA) Privacy Rule and Security Rule | 2000; 2003 | Covers protected health information (PHI) in any form; covers electronic protected health information (e-PHI) [9]. | Scope of covered entities often does not include most websites or phone apps, such as reproductive health apps, very often. “O[nly covers patient information](https://www.hhs.gov/ocr/privacy/hipaa/understanding/consumers/index.html) kept by health providers, insurers and data clearinghouses, as well as their business partners. HIPAA rules do not protect the privacy or security of individuals’ health information when they access or store it on personal cell phones or tablets” [10]. |
| Carpenter v. United States | 2018 | Established individual location protection identified through cell site location information [11]. | Do not cover IP address tracking as it is not considered an “exhaustive chronicle” of one's digital activities, and may not be traced directly to one specific user [12]. |
| California Consumer Privacy Act (CCPA) | 2018 | Secures new privacy rights for California consumers by providing users with control over the personal information that companies collect about them [13]. | Only applies to California residents and exempts HIPAA-eligible information which may be collected by reproductive health apps. Broad definition of 'business' and 'consumer’. Also only refers to for-profit businesses [14]. |
| Virginia Consumer Data Protection Act (VCDPA) | 2021 | Gives consumers the right to access their personal data, request its deletion, requires companies to conduct data protection assessments, and provides restrictions on data to directly identify individuals [15]. | User knowledge on options for data deletion may be limited. Companies may not state this explicitly as user data is used for analytics including marketing. Some reproductive health apps may fall under law exemptions. |
| California Privacy Rights Act (CPRA) | Approved in 2020; privacy protection beginning in 2023. | Amended CCPA giving users the right [to correct](https://oag.ca.gov/privacy/ccpa#sectione) inaccurate personal information that a business collects and [to limit](https://oag.ca.gov/privacy/ccpa#sectionf) the use and disclosure of sensitive personal information collected [16]. | Same limitations as CCPA with the main concern being the broad definition of ‘business’ and the inclusion of for-profit businesses only [13]. |
| *In the European Union (EU)* |  |  |  |
| EU General Data Protection Regulation (GDPR) | 2016 | Replaced the 1995 Data Protection Directive. Controls the processing of personal data which is “any information that relates to an identified or identifiable living individual (data subject)...” [17]. The legislation is meant to protect global users of EU based apps, regardless of the users location. | Does not equate to immunization from prosecution in the US. European companies more likely to comply with US authorities given legitimate legal requests. In addition “a European company may be hosting data outside the EU, making it subject to different legal frameworks and cross-border agreements” [18]. |

Due to the obsolescence of policies and their equivalent exclusion of reproductive health app data, local state officials are attempting to combat the threats to data safety and privacy posed by the use of electronic devices and technologies, and the loopholes associated with existing policies. The two policies that most closely address the issues faced with sharing of reproductive health data via reproductive health apps are as follows:

1. Fourth Amendment is Not for Sale Act introduced by U.S. Senators Ron Wyden, D-Ore., Rand Paul, R-Ky., and 18 other senators, that aims to “close the legal loophole that allows data brokers to sell Americans’ personal information to law enforcement and intelligence agencies without any court oversight” [19].
2. My Body, My Data Act of 2022 has been introduced in order to “establish protections, subject to certain limits such as provided written consent or necessity of information, for personal reproductive or sexual health information, including information relating to past, present, or future surgeries or procedures, such as the termination of a pregnancy.” It also sets out to establish what entities may or may not collect, retain, use, or disclose personal reproductive or sexual health information [20]. As of July 2023, this act has not been passed into legislation.

**References**

1. What does the Fourth Amendment Mean? United States Courts. Accessed July 25, 2023. https://www.uscourts.gov/about-federal-courts/educational-resources/about-educational-outreach/activity-resources/what-does-0#:~:text=The%20Constitution%2C%20through%20the%20Fourth,deemed%20unreasonable%20under%20the%20law.
2. Privacy and security. Federal Trade Commission: Protecting America’s Consumers . Accessed July 25, 2023. https://www.ftc.gov/business-guidance/privacy-security.
3. How to Determine if Your Product is a Medical Device. U.S. Food and Drug Administration. Accessed July 25, 2023. https://www.fda.gov/medical-devices/classify-your-medical-device/how-determine-if-your-product-medical-device.
4. Wetsman N. Birth control apps show the contradictions in FDA device oversight. The Verge. March 17, 2021. Accessed July 25, 2023. https://www.theverge.com/22335858/birth-control-app-clue-natural-cycles-fda.
5. Overview of Governmental Action Under the Stored Communications Act (SCA). Congressional Research Service. August 3, 2022. Accessed July 25, 2023. https://crsreports.congress.gov/product/pdf/LSB/LSB10801.
6. Kushner M. Stored Communications Act: The Good, the Bad, & the Ugly. Emden Law. September 9, 2015. Accessed July 25, 2023. https://www.emdenlaw.com/stored-communications-act-the-good-the-bad-and-the-ugly/#:~:text=Arguably%20the%20worst%20part%20of,obtain%20the%20information%20you%20need.
7. Children’s Online Privacy Protection Rule (“COPPA”). Federal Trade Commission. Accessed July 25, 2023. https://www.ftc.gov/legal-library/browse/rules/childrens-online-privacy-protection-rule-coppa.
8. The State of Play - Issue Brief: COPPA 101. Future of Privacy Forum. February 2, 2022. Accessed July 25, 2023. https://fpf.org/blog/the-state-of-play-issue-brief-coppa-101/.
9. Guide to Privacy and Security of Health Information. Department of Health and Human Services. Accessed July 25, 2023. https://www.healthit.gov/sites/default/files/pdf/privacy/onc_privacy_and_security_v1_022112.pdf.
10. Ornstein C. Federal Patient Privacy Law Does Not Cover Most Period-Tracking Apps. ProPublica: Investigative Journalism in the Public Interest . July 5, 2022. Accessed July 26, 2023. https://www.propublica.org/article/period-app-privacy-hipaa.
11. Supreme Court of the United States: CARPENTER v. UNITED STATES. The Supreme Court . Accessed July 25, 2023. https://www.supremecourt.gov/opinions/17pdf/16-402_h315.pdf.
12. Law Offices of John Wesley Hall. D.R.I.: IP addresses def used weren’t like detailed tracking information from CSLI requiring a SW. Casetext. November 5, 2018. Accessed July 25, 2023. https://casetext.com/analysis/dri-ip-addresses-def-used-werent-like-detailed-tracking-information-from-csli-requiring-a-sw?sort=relevance&resultsNav=false&q=.
13. Bonta R. California Consumer Privacy Act (CCPA). State of California - Department of Justice - Office of the Attorney General. May 10, 2023. Accessed July 25, 2023. https://oag.ca.gov/privacy/ccpa.
14. Jercich K. California Consumer Privacy Act can cause headaches for healthcare orgs. Healthcare IT News. September 21, 2021. Accessed July 26, 2023. https://www.healthcareitnews.com/news/california-consumer-privacy-act-can-cause-headaches-healthcare-orgs#:~:text=Challenges%20of%20data%20discovery%20and,compliance%20without%20an%20equitable%20ROI.
15. Virginia Consumer Data Protection Act (VCDPA): Everything you need to know about Virginia’s new comprehensive data privacy law. Bloomberg Law. Accessed July 25, 2023. https://pro.bloomberglaw.com/brief/virginia-consumer-data-protection-act-vcdpa/#:~:text=CCPAVCDPA%20Ambiguities-,What%20is%20the%20Virginia%20Consumer%20Data%20Protection%20Act%20(VCDPA)%3F,targeted%20advertising%20and%20sales%20purposes.
16. California Consumer Privacy Laws – CCPA & CPRA: Your guide to understanding the key provisions and impact of California’s comprehensive consumer privacy legislation – including enforcement, consumer rights, and how to comply. Bloomberg Law. Accessed July 25, 2023. https://pro.bloomberglaw.com/brief/california-consumer-privacy-laws-ccpa-cpra/.
17. European Union Country Commercial Guide: European Union - Data Privacy and Protection. Privacy Shield Framework. Accessed July 25, 2023. https://www.privacyshield.gov/ps/article?id=European-Union-Data-Privatization-and-Protection#:~:text=GDPR%20is%20a%20comprehensive%20privacy,for%20the%20movement%20of%20data.
18. Garamvolgyi F. Why US women are deleting their period tracking apps. The Guardian. June 28, 2022. Accessed July 25, 2023. https://www.theguardian.com/world/2022/jun/28/why-us-woman-are-deleting-their-period-tracking-apps.
19. Wyden, Paul and Bipartisan Members of Congress Introduce the Fourth Amendment Is Not For Sale Act. Ron Wyden United States Senator of Oregon. April 21, 2021. Accessed July 25, 2023. https://www.wyden.senate.gov/news/press-releases/wyden-paul-and-bipartisan-members-of-congress-introduce-the-fourth-amendment-is-not-for-sale-act-#:~:text=The%20Fourth%20Amendment%20is%20Not%20for%20Sale%20Act%20closes%20the,other%20businesses%20that%20have%20direct.
20. H.R.8111 - 117th Congress (2021-2022): My Body, My Data Act of 2022. Congress.gov. Accessed July 25, 2023. https://www.congress.gov/bill/117th-congress/house-bill/8111.
