# Supplementary material for: Exploration of Reproductive Health Apps’ Data Privacy Policies and the Risks Posed to Users: Qualitative Content Analysis
Source: J Med Internet Res. 2025 Mar 5;27:e51517. doi: 10.2196/51517 (PMC11923453; doi:10.2196/51517)
Supplement: Multimedia Appendix 2 [file jmir_v27i1e51517_app2.docx]

**Multimedia Appendix 2:** Privacy and data use practices of reproductive health apps from their respective data privacy policies collected in July 2023.

| **Reproductive health app** | **Policy accessibility** | **Data protection** | **Data collected and shared** | **Third-party data sharing** | **Opt-out option** | **Potential concerns** |
| --- | --- | --- | --- | --- | --- | --- |
| Clue [30] | Hyperlink within the app checkbox to create account; clickwrap approach | HTTPS protocol for encryption | Collects, stores, and uses some personal data about how users interact with their services, such as device data, event and use data, and IP address. Personal profile data are stored separately from cycle-tracking data and service settings and not collected from users aged <13 years. | ✓ | ✓ | Clearly states that data are shared with third parties, although said third parties do not use the data collected for anything other than Clue efficiency. However, it is easier for government agencies to obtain this information from said third parties rather than from the app itself. HTTP is vulnerable to third-party data attacks. |
| Flo [31] | Hyperlink within the app checkbox to create account; clickwrap approach | AWS^a^ KMS^b^ encryption | Collects technical identifiers (IP address, time zone, and mobile phone service provider), age group, subscription status, and fact of app launch. AppsFlyer is used for marketing and promotional purposes. Data are not collected from users aged <13 years. | ✗/✓^c^ | ✓ | Although the privacy policy states that data are not shared with third parties, AppsFlyer does share data with advertisement networks such as Facebook. The app also uses data processing services that collect user personal data. |
| Period Tracker by GP Apps [32] | Link to website checkbox to create account; clickwrap approach | ^--d^ | Vague description of data shared: “The Application obtains information you provide (i.e.., email address) when you register for an account and when you use the features of The Application.” | ✓Automatically collected data are shared; directly input data are said to be shared only under certain circumstances listed on the website | ✗/✓ | Little is done to protect user information. States that, because user sharing of data is voluntary, they are not responsible for any data collected by third parties. Specific third parties are not listed, unlike on other apps. Options not within the app include uninstalling the app or “Limit Ad Tracking” features through phone privacy and location services. |
| Stardust [33] | Landing page to website; no privacy policy checkbox: “By signing up you agree with our Terms of Use and Privacy Policy”; clickwrap approach | End-to-end encryption | Information shared to better user experiences and for analytics is explicitly written out, specifically stating what information is shared with what third parties, including registration, logins, and downloads from the app store. It does state that no PII^e^ is shared. All information is encrypted; IP address is not collected. | ✓ | ✓ | Stardust previously had issues with its privacy policy promises not aligning with their actions. The privacy policy link is written in small font under the registration; difficult to see due to the small font and color chosen. |

^a^AWS: Amazon Web Services.

^b^KMS: Key Management Service.

^c^✗/✓ signifies the partial presence of a feature. For example, an app may state that there is no third-party data sharing; however, it may also state that the analytic services they use can share data with other third parties.

^d^Not available.

^e^PII: personal identifiable information.

**References**

30. Clue Privacy policy. Clue. May 11, 2023. Accessed July 23, 2023. https://helloclue.com/privacy.

31. Flo Privacy policy. Flo Health. September 14, 2022. Accessed July 25, 2023. https://flo.health/privacy-policy.

32. Privacy Policy. GP Apps. Accessed July 25, 2023. https://gpapps.com/support/privacy-policy/.

33. We do not and will not sell your data. Period. Stardust. Accessed July 25, 2023. https://stardust.app/privacy-policy.html.
